# Basic Fibroblast Growth Factor-Anchored Multilayered Mesenchymal Cell Sheets Accelerate Periosteal Bone Formation

**DOI:** 10.1155/2017/4371460

**Published:** 2017-07-06

**Authors:** Kentaro Uchida, Gen Inoue, Osamu Matsushita, Kyosuke Horikawa, Hiroyuki Sekiguchi, Wataru Saito, Shotaro Takano, Hisako Fujimaki, Masayuki Miyagi, Masashi Takaso

**Affiliations:** ^1^Department of Orthopaedic Surgery, Kitasato University School of Medicine, Sagamihara, Kanagawa, Japan; ^2^Department of Bacteriology, Okayama University Graduate School of Medicine, Dentistry and Pharmaceutical Sciences, 2-5-1 Shikata-cho, Kita-ku, Okayama, Japan; ^3^Okayama University Medical School, 2-5-1 Shikata-cho, Kita-ku, Okayama, Japan

## Abstract

Cell-based regenerative therapy has the potential to repair bone injuries or large defects that are recalcitrant to conventional treatment methods, including drugs and surgery. Here, we developed a multilayered cell-based bone formation system using cells coated with fibronectin-gelatin (FN-G) nanofilms. The multilayered mesenchymal cells (MLMCs) were formed after two days of culture and were shown to express higher levels of BMP-2 and VEGF compared to monolayer cultures of MCs. The MLMCs were used as a graft material in combination with a fusion protein consisting of basic fibroblast growth factor (bFGF), polycystic kidney disease (PKD) domain, and the collagen-binding domain (CBD) of* Clostridium histolyticum* collagenase. In femur sites grafted with the MLMCs, significantly higher levels of callus volume and bone mineral content were observed compared to the sham controls. The callus volume and bone mineral content were further increased in femur sites grafted with bFGF-PKD-CBD/MLMCs. Taken together, these results suggest that bFGF-PKD-CBD/MLMCs, which can be simply and rapidly generated in vitro, have the potential to promote bone repair when grafted into large defect sites.

## 1. Introduction

Cell-based regenerative therapy has the potential to repair injured or defect-containing bone that is resistant to conventional medical treatments, including growth-stimulating drugs and surgeries. Mesenchymal stem cells (MSCs) are an attractive autologous source of somatic stem cells for cell-based bone regenerative therapy, as they proliferate actively in vitro and differentiate into bone cells [1–5]. Several types of layered cell technologies, such as cell sheets constructed in temperature-responsive culture dishes [6–8], magnetic liposomes [9, 10], and cell-containing gel layers [11], have been applied towards the treatment of injured tissues. Although these approaches accelerate tissue healing, the cells may contain intracellular magnetic particles, which may have adverse effects.

To overcome the limitations of cell-layering techniques developed to date, a simple and rapid tissue engineering approach for generating multilayered cells was developed using fibronectin-gelatin (FN-G) nanofilms [12]. This cell-accumulation technique allowed for mouse fibroblast cells to form approximately eight layers in vitro after a 24 h incubation. MSCs secrete trophic factors and accelerate wound healing compared to fibroblasts [13]. Due to these promising results, this method may also be applicable for forming multiple layers of MSCs for use in bone grafting. However, although the cell-accumulation technique has been evaluated in vitro with murine fibroblasts, the potential of this system to promote bone repair in vivo has not been investigated with MSCs.

The exogenous application of growth factors, particularly basic fibroblast growth factor (bFGF), has been shown to promote tissue regeneration when performing bone grafting [14–17]. bFGF is a potent mitogen for MSCs and promotes angiogenesis [18, 19], bone formation [20–24], and nerve regeneration [25, 26]. We previously demonstrated that the subcutaneous injection of a recombinant protein consisting of the polycystic kidney disease (PKD) and* Clostridium histolyticum* collagenase collagen-binding domains (CBD) fused to basic fibroblast growth factor (bFGF; bFGF-PKD-CBD) had greater skin fibroblast growth-promoting effects in nude mice than native bFGF [27]. More recently, bFGF-PKD-CBD was shown to enhance bone formation at lower concentrations than bFGF alone when loaded onto implantable collagen sheets [28, 29], suggesting that the treatment combination of bFGF-PKD-CBD and a multilayered cell construct consisting of MSCs may promote ectopic bone formation at defect sites.

Here, we constructed multilayered mesenchymal cell (MLMCs) sheet anchored to collagen-binding bFGF using a novel cell tissue engineering technique. The properties and bone formation capacity of this material were evaluated both in vitro and in vivo using a rat femur model.

## 2. Materials and Methods

### 2.1. Isolation of Rat Mesenchymal Cells

A specific pathogen-free colony of Sprague-Dawley rats was housed in a semibarrier system with a controlled environment (temperature, 23 ± 2°C; humidity, 55%  ±  10%; and lighting, 12 h light/dark cycle) at Nippon Charles River Laboratories (Kanagawa, Japan) and were fed a diet of standard rodent chow (CRF-1; Oriental Yeast Co., Ltd., Tokyo, Japan). The periosteum of distal femurs harvested from 10-week-old male rats, as previously described [28, 30], was used for the isolation of nucleated periosteal cells, which were then plated at 1 × 10^4^ cells/cm^2^ in 6-well culture plates containing *α*-minimum essential medium (*α*-MEM) supplemented with 10% fetal bovine serum, 100 U/ml penicillin, and 100 *μ*g/ml streptomycin. The cells (passage 0 [P0]) were incubated at 37°C in 5% CO_2_ for 7 days and expression of the mesenchymal cell markers CD29, CD54, and CD90 was confirmed by flow cytometry using antibodies against CD45 (fluorescein isothiocyanate, FITC), CD29 (phycoerythrin, PE), CD54 (PE), and CD90 (peridinin chlorophyll protein, PERCP; Biolegend, San Diego, CA, USA) [30]. All animal procedures were performed in accordance with the approval of the animal ethics committee of Kitasato University.

### 2.2. Preparation of bFGF and CB-bFGF

Recombinant human bFGF was purchased from Kaken Pharmaceuticals (Tokyo, Japan). bFGF-PKD-CBD was produced using recombinant techniques, as described previously [28, 29].

### 2.3. Preparation of Multilayered Mesenchymal Cell Constructs

For the construction of MLMCs, FN-G nanofilms were first prepared on single-cell surfaces using a layer-by-layer assembly approach with a multilayered cell culture kit (Cell Feuille, Sumitomo Bakelite Co., Ltd., Tokyo, Japan). Briefly, 5 × 10^6^ cells/ml of P2 rat mesenchymal cells collected by centrifugation at 300*g* after trypsinization, were alternatively incubated with 0.2 mg/mL FN (Mw 4.6 × 10^5^) and G (Mw 1.0 × 10^5^) in 50 mM Tris-HCl (pH 7.4) for 1 min at room temperature with mixing at 30 rpm using a Microtube Rotator (TAITEC Co., Saitama, Japan). After each treatment, the cells were washed with 50 mM Tris-HCl (pH 7.4) using centrifugation at 200*g* for 1 min to remove unadsorbed polymers. After five cycles of the immersion steps, the FN-G nanofilms were coated onto the cell surfaces. A total of 2 × 10^6^ cells coated in the FN-G nanofilm were seeded into a cell culture insert coated with a FN and were further incubated in *α*-MEM containing 10% FBS at 37°C. Noncoated cells were used as controls. After two days of incubation, the histology and mRNA expression profiles of layered cells were evaluated by cryosection and real-time PCR, respectively, as described below.

### 2.4. Histology of MLMCs

After two days of incubation, noncoated and FN-G nanofilm-coated MLMCs were fixed in 4% paraformaldehyde at 4°C for 48 h and were then immersed in a carboxymethyl cellulose (CMC) gel, transferred to hexane, and completely frozen using solid CO_2_. The frozen samples were cut into 6 *μ*m thick sections using a CM 3050S IV cryomicrotome (Leica Instruments, Germany). After drying, the sections were stained with hematoxylin and eosin (HE) using standard procedures (Kureha Special Laboratory Co., Tokyo, Japan).

### 2.5. Real-Time PCR

Total RNA was extracted from monolayer and multilayered mesenchymal cells using TRIzol reagent (Invitrogen, Carlsbad, CA) according to the manufacturer's instructions and was used as template for first-strand cDNA synthesis using SuperScript III RT (Invitrogen). The PCR reaction mixture consisted of 2 *μ*L cDNA, specific primer set (0.2 *μ*M final concentration), 12.5 *μ*L SYBR Premix Ex Taq (Takara, Kyoto, Japan), and an appropriate amount of ultrapure sterile, deionized water to give a final volume of 25 *μ*L. The sequences of the PCR primer pairs used in this study are listed in Table 1. Quantitative PCR was performed using a Real-Time PCR Detection System (CFX-96; Bio-Rad, CA, USA) and the following PCR cycle parameters: denaturation at 95°C for 1 min, followed by 40 cycles of 95°C for 5 sec and 60°C for 30 sec. mRNA expression of the target genes was normalized to the levels of GAPDH mRNA (*n* = 8).

### 2.6. Bone Formation Ability of Multilayered Mesenchymal Cell Construct In Vivo

To investigate the bone formation ability of MLMCs in vivo, FN-G nanofilm-coated MLMCs were seeded onto culture inserts, which contain an atelocollagen membrane on the anterior surface (Koken Co., Ltd., Japan). After two days of incubation at 37°C in 5% CO_2_, the collagen membranes with MLMCs were cut out from the bottom of the culture insert and were further incubated with or without 0.58 nmoles CB-bFGF solutions at 4°C for 30 min. To prepare rat femurs for receiving the MLMC grafts, leg hair was shaved and a skin incision was then made in the center of the thigh to expose the right femur of the hind leg. MLMCs were then grafted onto the periosteum of the anterior surface of the exposed femur. Sham operated rats without grafting were used as controls. After surgery, the rats were allowed to use their hind legs without restriction. To assess new bone formation, 8 rats in each group were sacrificed with excess CO_2_ gas 7 days after performing the MLMCs grafting.

### 2.7. Quantification of New Bone Volume and Bone Mineral Content

Seven days after performing the MLMCs grafts, rats were sacrificed, and the femurs and surrounding muscle were excised. The femurs were placed in 4% paraformaldehyde, stored at 4°C for 48 h, and then immersed in PBS. Micro-CT images of the graft regions were captured using a Microfocus X-ray CT system (inspeXio SMX-90CT; Shimadzu Co., Ltd., Tokyo, Japan) and the following settings: acceleration voltage, 90 kV; current, 110 mA; voxel size, 20 *μ*m/pixel; and matrix size, 1024 × 1024. Images of the entire femur were first obtained, and 10 mm regions of interest (500 slices) were defined at the midfemur. New bone volume and bone mineral content were measured using three-dimensional (3D) image analysis software (Tri-3D-Bon; Ratoc System Engineering Co., Ltd., Tokyo, Japan) as previously described [28, 31].

## 3. Results

### 3.1. Preparation of MLMC Constructs

**Figure 1:**
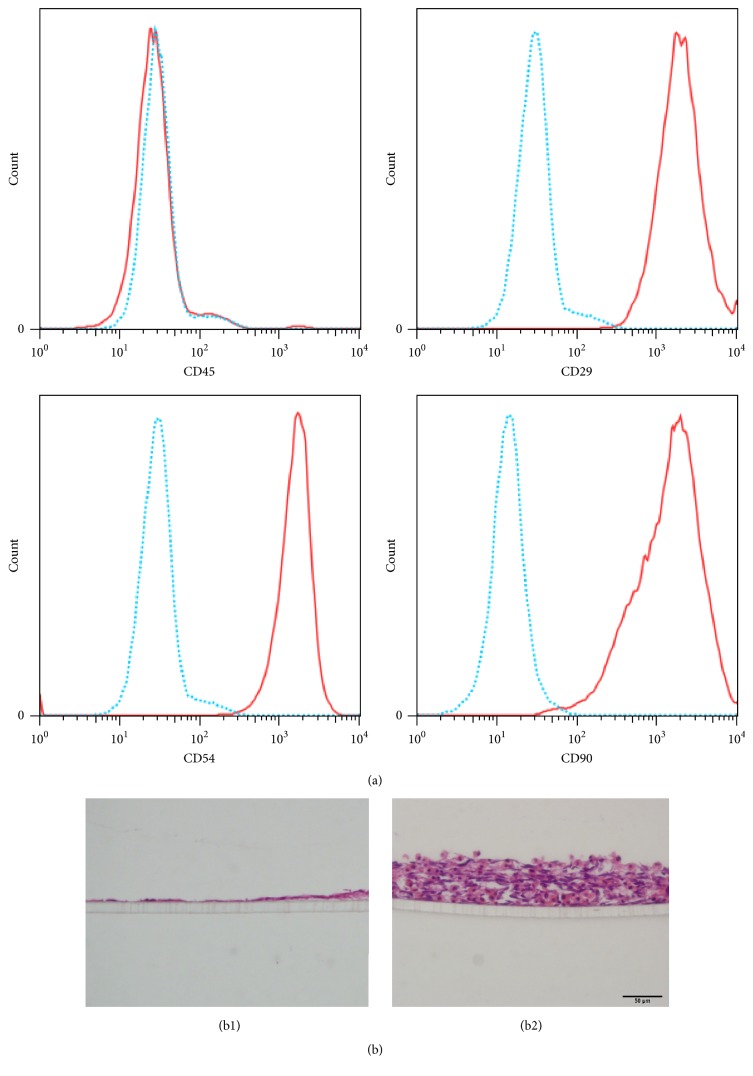
*Construction of multilayered tissues using a cell-accumulation technique with fibronectin-gelatin (FN-G) nanofilms*. (a) Analysis of cell-surface markers in isolated periosteal mesenchymal cells. Blue: nonstaining control. Red: stained sample. (b) Hematoxylin-eosin (HE) staining of the multilayered mesenchymal cells. (b1) non-FN-G-coated cells and (b2) FN-G-coated cells. Scale bar = 50 *μ*m.

Isolated periosteal cells were positive for the mesenchymal stem cell markers CD54 and CD29 and CD54 and CD90 and were negative for the hematopoietic cell marker CD45 (Figure 1(a)). After two days of culture, the non-FG-N-coated cells formed a single layer on the cell culture plate inserts. In contrast, the FG-N-coated periosteal mesenchymal cells clearly formed a layer with an average thickness of eight cells (Figure 1(b)).

### 3.2. Gene Profile of MLMCs

**Figure 2:**
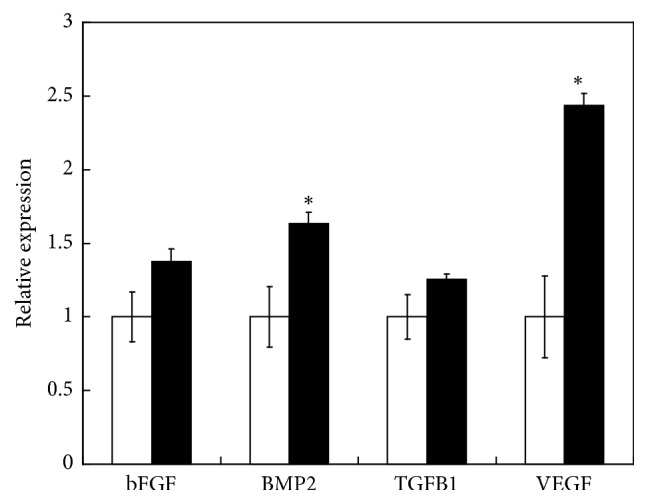
*Gene expression of trophic factors in multilayered cells*. Results of real-time PCR analysis of bFGF, BMP-2, TGF-*β*1, and VEGF mRNA expression in non-FN-G-coated (white) and FN-G-coated cells (black). *∗* indicates a statistically significant difference between the coated and noncoated cells. All data are presented as the mean ± standard error (*n* = 8).

Mesenchymal stem cells secrete trophic factors such as VEGF, TGF-b, BMP-2, and bFGF, which contribute to musculoskeletal regeneration in cell therapies [32–37]. Therefore, to assess the potential contribution of MLMC grafts to bone regeneration, we examined trophic factor gene expression in MLMCs. The expression levels of BMP-2 and VEGF in the MLMCs were significantly higher than those in monolayers of mesenchymal cells (Figure 2). In contrast, no differences in the expression of bFGF or TG*β*1 were detected between the multilayered and monolayer mesenchymal cells.

### 3.3. In Vivo Periosteal Bone Formation by MLMCs and CB-bFGF-Anchored MLMCs

**Figure 3:**
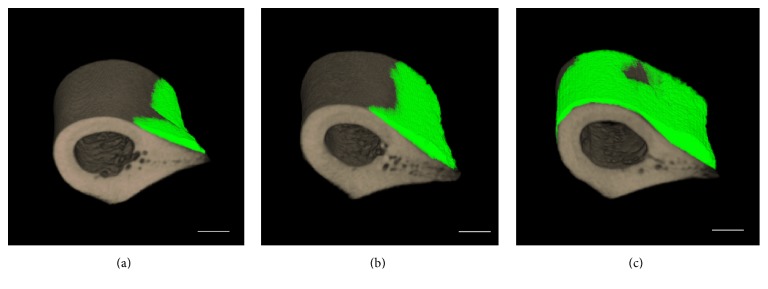
*3D micro-CT analysis of femurs after the grafting of multilayered mesenchymal cells loaded with bFGF-PKD-CBD*. 3D micro-CT images of fractured rat femurs treated with (a) sham, (b) MLMCs, and (c) MLMCs/bFGF-PKD-CBD after two weeks of recovery. Green: newly formed bone; gray: existing bone. Scale bars indicate 3 mm.

**Figure 4:**
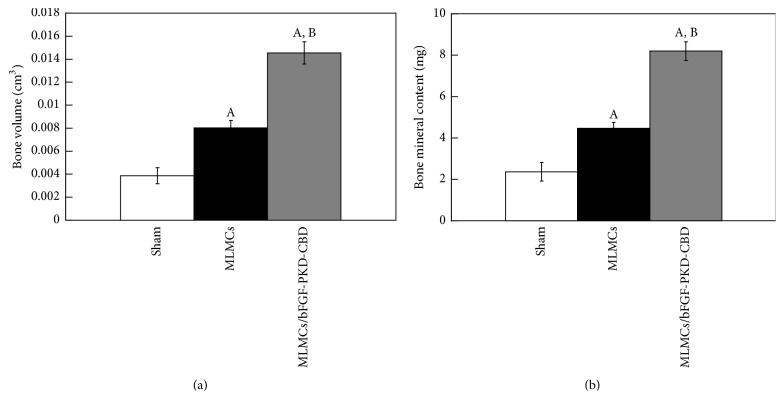
*Properties of new bone formed in rat femurs grafted with multilayered mesenchymal cell loaded with CB-bFGFs*. (a) Callus volume (cm^3^) and (b) bone mineral content (mg). Data are presented as the mean ± SE (*n* = 8). ^A^*P* < 0.05 compared with the sham group. ^B^*P* < 0.05 compared with the MLMC group.

Periosteal bone formation in rat femurs subjected to a sham operation or grafted with MLMCs either alone or in combination with CB-bFGF (CB-bFGF/MLMCs) was evaluated by micro-CT image analysis one week after grafting. In femurs grafted with MLMCs and CB-bFGF/MLMCs, higher levels of bone formation and bone mineral content were observed compared to the femurs of sham operated rats (Figures 3, 4(a), and 4(b)). Notably, the new bone volume and bone mineral content were significantly higher in femurs grafted with CB-bFGF/MLMCs compared to the femurs grafted with MLMCs alone (Figures 3, 4(a), and 4(b)).

## 4. Discussion

To develop a novel and effective method for cell-based regenerative therapy, rat mesenchymal cells were coated with FN/G to promote the rapid formation of cell layers. The resulting MLMCs had higher expression levels of BMP-2 and VEGF compared to monolayer-cultured mesenchymal cells. When grafted into rat femur sites, the MLMCs promoted significantly higher callus formation and the resulting bone had a higher mineral content compared to the bone of the sham controls. Further increases in bone formation and mineral content were observed in femur sites grafted with MLMCs combined with the recombinant protein CB-bFGF. Taken together, the results of the present study suggest that the CB-bFGF/MLMC construct, which can be simply and rapidly generated in vitro, may have the potential to promote bone formation in a clinical setting.

Cell sheet technologies have been successfully developed and used for bone regeneration in several animal models [38, 39]. For example, the transplantation of monolayer-cultured mesenchymal stem cell sheet enhances bone formation in a rat nonunion model [38]. Similarly, mesenchymal stem cell sheets constructed using magnetite nanoparticles stimulated ossification in rat crania [39]. To overcome inherent problems like the fact that nonbiocompatible materials, such as magnetic particles, remain intracellular, here, multilayers of periosteal mesenchymal cells were constructed using an FN-G nanofilm-based technique. We confirmed that the MLMCs were formed after only two days of culture and further demonstrated that transplantation of the MLMCs into rat femurs promoted bone formation. These results suggest that the coating of MSCs with FN-G nanofilms promoted multicell layer formation without leaving residual nonbiocompatible materials and that this tissue engineering technique may be useful for bone regenerative therapy.

Therapeutic cells such as MSCs, embryonic stem cells and endothelial cells secrete numerous trophic factors that contribute to tissue repair [34–37, 40–42]. For example, MSC-conditioned medium accelerates osteogenesis in a distraction osteogenesis mouse model [40], and endothelial cell-produced BMP-2 promotes the osteogenic differentiation of MSCs. Further, the secretion of VEGF by MSCs on a hydroxyapatite/poly(lactide-co-glycolide) scaffold was speculated to contribute to osteogenesis when the cellular construct was transplanted into the dorsum of nude rats [35]. In addition, the production of VEGF and the observed osteogenic effects were shown to be dependent on the composition of the scaffold. Here, the expression levels of BMP-2 and VEGF were increased in MLMCs compared to monolayer mesenchymal cells. Consistent with this finding, MLMCs stimulated periosteal bone formation in vivo.

To enhance osteogenic activity, the transplantation of MSCs combined with growth factors, particularly bFGF, has been investigated [16, 43]. bFGF has anabolic effects in the process of bone regeneration and also stimulates MSCs proliferation [20–23]. MSCs have been used as vectors for bFGF gene transfer and local drug delivery to promote bone regeneration following distraction osteogenesis in a rabbit model [16]. However, a major concern of gene therapy is the ability to control transgene expression, as the overexpression of growth factors may lead to adverse side effects [44]. We previously established a sustained release system consisting of collagen materials coated with a recombinant bFGF containing a PKD and CBD derived from* C. histolyticum* ColH and demonstrated that this system accelerates periosteal bone formation and growth in bone fracture and defect models [28–30, 45]. Here, the CB-bFGF/MLMC constructs stimulated periosteal bone formation in a rat femur to a significantly higher extent compared to MLMCs alone, suggesting that the CB-bFGF/MLMC constructs are promising materials for accelerating bone formation in clinical settings.

bFGF and BMP-2 promote mesenchymal proliferation and osteogenic differentiation during bone healing, respectively [46]. Previous studies also reported that the combination of bFGF and BMP2 synergistically promote bone formation [46–48]. For example, the combination of BMP-2 and bFGF enhances osteoblastic differentiation of cultured rat bone marrow mesenchymal stromal cells in vitro and synergistically promotes ectopic bone formation in vivo [46]. In addition, coadministration of bFGF and BMP-2 promotes cranial [47] and mandibular defects [48]. These findings suggest that the bone forming ability of CB-bFGF/MLMC constructs may result from the actions of bFGF- and MLMC-secreted BMP-2 to stimulate proliferation and promote differentiation, respectively.

Several limitations of the present study warrant mention. First, we did not evaluate whether the CB-bFGF/MLMC construct has the potential to promote bone repair when grafted into large defect sites. Second, whether MSCs generated using this system differentiate into bone in the transplanted site in vivo remains to be determined. Cell tracing should be performed to evaluate the fate of MSCs. Finally, we used rat mesenchymal cells. Further investigation using human mesenchymal cells is needed to examine the potential of CB-bFGF/MLMC constructs in clinical settings.

In conclusion, we developed a novel and simple system for promoting bone formation by combining MLMCs and a bFGF-PKD-CBD fusion protein. Compared to MLMCs alone, the MLMCs/bFGF-PKD-CBD construct significantly increased callus volume and bone mineral content at the fracture sites within two weeks of grafting. These findings suggest that the MLMC/bFGF-PKD-CBD composite is a promising and relatively simple method for promoting bone formation in a clinical setting.

## Figures and Tables

**Table 1:** Sequences of the primers used in this study.

Gene	Direction	Primer sequence (5′-3′)	Product size (bp)
bFGF	F	TCC AAG CAG AAG AGA GAG GA	205
	R	TGC CCA GTT GGT TTC AGT G	
BMP2	F	AAG GCA CCC TTT GTA TGT GG	211
	R	GCT AAG CTC AGT GGG GAC AC	
TGF*β*1	F	GAC CGC AAC AAC GCA ATC TA	110
	R	GAC AGC AAT GGG GGT TCT GG	
VEGF	F	GTA CCT CCA CCA TGC CAA GT	231
	R	CAC TCC AGG GCT TCA TCA TT	
GAPDH	F	TGC CAC TCA GAA GAC TGT GG	129
	R	TTC AGC TCT GGG ATG ACC TT	
